# Aberrant Brain Regional Homogeneity and Functional Connectivity of Entorhinal Cortex in Vascular Mild Cognitive Impairment: A Resting-State Functional MRI Study

**DOI:** 10.3389/fneur.2018.01177

**Published:** 2019-01-22

**Authors:** Meimei Zuo, Yi Xu, Xiaomin Zhang, Man Li, Xiuqin Jia, Jinliang Niu, Dongfang Li, Yanqing Han, Yanhui Yang

**Affiliations:** ^1^Medical Department, Cangzhou People's Hospital, Cangzhou, China; ^2^Department of Image, The Second Affiliated Hospital of Shanxi Medical University, Taiyuan, China; ^3^Department of Neurology, The Second Affiliated Hospital of Shanxi Medical University, Taiyuan, China; ^4^Department of Radiology, Beijing Chaoyang Hospital, Capital Medical University, Beijing, China; ^5^Department of Radiology, Xuanwu Hospital, Capital Medical University, Beijing, China; ^6^Beijing Key Laboratory of Magnetic Resonance Imaging and Brain Informatics, Beijing, China

**Keywords:** vascular mild cognitive impairment, entorhinal cortex, resting-state functional MRI, regional homogeneity, functional connectivity

## Abstract

The aim of this study was to investigate changes in regional homogeneity (ReHo) and the functional connectivity of the entorhinal cortex (EC) in vascular mild cognitive impairment (VaMCI) and to evaluate the relationships between such changes and neuropsychological measures in VaMCI individuals. In all, 31 patients with VaMCI and 32 normal controls (NCs) underwent rs-fMRI. Differences in whole-brain ReHo and seed-based bilateral EC functional connectivity (EC-FC) were determined. Pearson's correlation was used to evaluate the relationships between regions with significant group differences and different neuropsychological measures. Vascular mild cognitive impairment (VaMCI) patients had lower scores in Mini-mental State Examination (MMSE) and Montreal Cognitive Assessment (MoCA) and higher ones in Activity of Daily Living (ADL) (*p* < 0.05). Vascular mild cognitive impairment (VaMCI) individuals had significantly lower ReHo in the left cerebellum and right lentiform nucleus than NCs (*P* < 0.05, TFCE FWE correction). Vascular mild cognitive impairment (VaMCI) subjects showed significant decreases in the FC of the right EC in the right inferior frontal gyrus, right middle frontal gyrus, bilateral pre-central gyrus, and right post-central/superior parietal lobules (*P* < 0.05, TFCE FWE correction). Significant positive correlations were found between ReHo and MoCA scores for the right lentiform nucleus (*r* = 0.37, *P* < 0.05). The right post-central/superior parietal lobules showed a significant positive correlation between right EC-FC and MoCA scores (*r* = 0.37, *P* < 0.05). Patterns in ReHo and EC-FC changes in VaMCI patients and their correlations with neuropsychological measures may be a pathophysiological foundation of cognitive impairment, which may aid the early diagnosis of VaMCI.

## Highlights

- We investigated spontaneous neural activity and the functional connectivity of the entorhinal cortex (EC) in VaMCI patients.
- The VaMCI individuals had significantly lower ReHo in the left cerebellum and right lentiform nucleus.
- The VaMCI subjects showed significant decreases in the FC of the right EC in the right inferior frontal gyrus, right middle frontal gyrus, bilateral pre-central gyrus and right post-central/superior parietal lobules.
- ReHo and EC-FC changes and their correlations with neuropsychological measures may aid the early diagnosis of VaMCI.

## Introduction

Dementia is a public health issue (1, 2). Vascular dementia is now recognized as the second most common form of dementia after Alzheimer's disease, and there is increasing awareness that early intervention may help prevent dementia, even of the Alzheimer type (1, 3, 4). Therefore, the early identification of vascular cognitive impairment (VCI), particularly vascular mild cognitive impairment (VaMCI), is particularly important.

Resting state functional MRI (rs-fMRI), which has been established as a useful non-invasive technique for determining how structurally segregated and functionally specialized cerebral centers are interconnected, has been receiving increased attention in brain science research in recent years and is widely used in the study of diseases of cognitive impairment (5–7). A valuable method for analyzing the local features of rs-fMRI signals (8), regional homogeneity (ReHo) measures the temporal synchronization of time series of nearest neighbors and can be used to map local spontaneous neural activity, making it a useful tool for identifying changes in cerebral activity (7–9). Functional connectivity (FC) can be used to map long-distance connectivity and to detect the undiscovered haemodynamic responses that ReHo cannot reveal (7).

ReHo has also been used in several clinical studies, including attention deficit hyperactivity disorder (ADHD), AD, and MCI (10, 11). Zhang et al. report that the patients with MCI exhibit altered ReHo in the medial prefrontal cortex, the bilateral posterior cingulate gyrus/precuneus and the left inferior parietal lobule (IPL), and higher of ReHo in the left IPL could indicate the presence of a compensatory mechanism in MCI (11). For FC analysis, the choice of regions of interest (ROI) is not always the same previous studies mainly focus on the posterior cingulate cortex (PCC) connectivity and its crucial role in cognitive function and memory. Ding et al. report that patients with subcortical VCI (sVCI) exhibit decreases in FC of the left thalamus with the PCC (12). In addition, Deng et al. found that the patients with VaMCI exhibited altered ReHo in ACC-FC in some regions, and decreases in the left pre-central gyrus (13). Wang et al. and Li et al. selected the thalamus and Meynert basal nuclei (BNM), respectively, as the seed voxel and demonstrated a markedly abnormal FC mode in mild cognitive impairment (MCI) (5, 14). Less attention has been devoted to the potential role of brain regions that are significantly related to cognitive function, such as the entorhinal cortex (EC).

EC is the gate for multimodal information from many cortices, which converges onto the hippocampus. The EC–hippocampal neural network is the key center for learning, episodic memory and performing spatial navigation (15). Hafting et al. found grid cells with strong discharge on specific location of space in EC, and the specific anatomical basis determined the relative specificity of spatial memory (16). Von Gunten et al. found that alteration starts from the EC and then gradually expanded to the hippocampus and surrounding structures from MCI to AD (17, 18). According to previous studies, the EC, a vital region for widespread cognitive function, was defined as ROI in the current study. We hypothesized that the ReHo and EC functional connectivity (EC-FC) would be disrupted in VaMCI patients.

## Materials and Methods

### Subjects and Assessments

All participants were given a detailed explanation of the study and signed an informed consent form prior to its commencement. During the selection of subjects, all of the subjects were performed T1-weighted image (T1WI), T2-weighted image (T2WI), diffusion weighted imaging (DWI), fluid-attenuated inversion-recovery (FLAIR), and rs-fMRI scanning routinely.

In all, 31 right-handed VaMCI patients were recruited from among outpatients and inpatients of the neurology department of the Second Affiliated Hospital of Shanxi Medical University from January 2017 to December 2017. The inclusion criteria for the VaMCI group were based on the statement of the Society for Vascular Behavioral and Cognitive Disorders and the diagnostic guidelines for VaMCI in China, which include the following (19, 20): there exist risk factors for cerebrovascular disease or cerebral vascular diseases such as hypertension, diabetes mellitus, hyperlipemia, or others; imaging examination revealed evidence of cerebrovascular lesions such as white matter lesions in key infarcts and multiple lacunar infarcts. In addition, we took a coronal scanning to exclude the patients with hippocampal atrophy; patients themselves or their families complain of a decline in cognition, with such symptoms of cognitive decline lasting for at least 6 months and having a fluctuating course; damage to cognitive function and risk factors of cerebrovascular disease are directly related to cerebrovascular disease; activity of daily living (ADL) is normal or near normal with a ADL score < 26; cognitive abilities is normal, with a Mini-mental State Examination (MMSE) score ≥24; Montreal Cognitive Assessment (MoCA) score < 26; Hachinski Ischemic Score (HIS) ≥7; Clinical Dementia Rating (CDR) = 0.5; and cognitive impairment has not yet reached the standard of a diagnosis of dementia in the Diagnostic and Statistical Manual of Mental Disorders, Fourth Edition (DSM-IV).

From the community, 32 right-handed NCs were recruited for comparison. The inclusion criteria for the NCs were as follows: no current or previous diagnosis of any neurological or psychiatric disorders; no neurological deficiencies in physical examinations; absence of abnormal findings on brain MRI; no complaints of cognitive changes; and a CDR = 0. Additional exclusion criteria for both VaMCI and NCs participants included contraindications for MRI such as use of cardiac pacemakers and claustrophobia (21).

A medical history was taken on all participants, who also received physical examinations and neuropsychological tests. MMSE was used to assess patient status in cognitive abilities in five aspects, including orientation, memory, attention, computation power, and language competence. The level of education for all participants was required to be junior high school and above. MoCA was used to assess cognitive abilities in the following eight aspects: visual space, executive function, naming, attention, language, abstract, delayed recall, and directional force. An MoCA score < 26 points means that the patient's cognitive function is damaged. ADL was used to assess the patients' activities of daily living. CDR was used to assess the degree of cognitive impairment of patients. HIS was used to differentiate the nature of the cognitive impairment. An HIS score ≥7 points means that the patient's cognitive impairment is caused by vascular factors. The Hamilton Anxiety Rating scale (HAMA) and Hamilton Depression Rating scale (HAMD) were used to assess the patients' activity.

### MRI Acquisition

MRI data were acquired with a 3-Tesla Trio scanner (General Electric Discovery silent, MR750W, America). All participants were asked to lie still, with their eyes closed. Foam padding was employed to limit head motion, and headphones were used to reduce scanner noise. Functional images were acquired using an echo-planar imaging (EPI) sequence with a repetition time (TR)/echo time (TE)/flip angle (FA) = 2,000 ms/30 ms/90, field of view (FOV) = 220 × 220 mm, slice thickness/gap = 3.6/0.4 mm and a data matrix = 64 × 64.

### Image Data Pre-processing and Analysis

Rs-fMRI data were pre-processed and analyzed using statistical parametric mapping software (SPM12, http://www.fil.ion.ucl.ac.uk/spm) and the data processing assistant for rs-fMRI (DPARSF V 4.3, http://www.restfmri.net), created using Matlab R2014a. The original data from the Digital Imaging and Communications in Medicine (DICOM) format were converted into a Neuroimaging Informatics Technology Initiative (NIFTI) format using SPM12 software (22). The first 10 functional images were discarded to reduce the fluctuation of MRI signals during the initial stages of scanning. To correct all layers to the same point, different time points among layers in the course of scanning needed to be corrected. It took several minutes to scan the functional images, so the subjects would inevitably move their heads, caused by breathing, blood flow, or other physiological factors. The geometric displacement caused by the head motion was corrected: data from subjects whose head translation was more than 2 mm in any direction (x, y, or z) or whose angle of head rotation was >2° were deleted in the process of scanning. To compare the images of different subjects using the same method, each participant's image was converted into a standard size and orientation. All subjects' functional images were registered onto an EPI cerebral template (23). Then these image data were resampled based on a 3 × 3 × 3 mm^3^ volume unit, and all functional data were standardized in Montreal Neurological Institute (MNI) space through parameter conversion. To reduce baseline drift caused by noise, the linear trend was removed after functional images were spatially normalized. To reduce physiological interference, all functional images were processed using low-frequency filtering, from 0.01 to 0.08 Hz. A linear regression model was used to further remove the interference of other possible influencing factors, such as head and white-matter cerebrospinal fluid. The ReHo calculation was performed using pre-processed images, and the resulting images were smoothed with an isotropic Gaussian kernel of 4 mm full-width half-maximum (8). FC methods based on low-frequency (0.01–0.08 Hz) spontaneous blood oxygenation level-dependent (BOLD) fluctuations in rs-fMRI provide a powerful tool for characterizing intrinsically functional associations among brain regions. In this study, the bilateral EC ROI were generated using the free Anatomy Toolbox software V2.2b (22). For each seed region, the BOLD time series of the voxels within the seed region was averaged to generate a reference time series. To further enhance the normality of the data analysis, the correlation r value was transformed into a *z*-value using the Fisher *r*-to-*z* transformation.

## Statistical Analysis

The statistical software package SPSS 19.0 was used to compare demographic and Neuropsychological measures. A two-sample *t*-test was adopted to compare the age, education and neuropsychological measures between the VaMCI and NC groups. The chi-square test was used to compare sex differences between groups. The difference was statistically significant when *P* < 0.05.

A one-sample *t*-test was used to acquire the mode patterns of ReHo and EC-FC using the statistical analysis tool REST (8, 22). A result was considered statistically significant at *P* < 0.05 (threshold free cluster enhancement family wise error, TFCE FWE corrected) and voxels >10. A two-sample *t*-test was used to compare differences in ReHo and EC-FC between VaMCI and NC. A *P* < 0.05 and cluster size threshold >10 voxels was considered statistically significant (FDR corrected). The XjView software package (http://www.alivelearn.net/xjview) was used to confirm the specific anatomical positions corresponding to the MNI coordinates, which were statistically significant for the brain region.

ROI analysis was performed on the regions showing significant ReHo or EC-FC changes in patients with VaMCI compared to NCs. Partial correlations were conducted to evaluate the relationship between abnormal functional changes demonstrated by ReHo or EC-FC values in these ROIs and neuropsychological assessments in patients with VaMCI. Statistical significance was set at *p* < 0.05.

## Results

### Demography and Neuropsychological Tests

Demographic characteristics and neuropsychological scores are shown in Table 1. There were no significant differences between the two groups in sex, age, years of education, the degree of anxiety, or depression. Compared to the NC group, the VaMCI patients had lower scores in MMSE (*p* < 10^−4^) and MoCA (*p* < 10^−4^) and greater ADL (*P* = 0.001) scores.

**Table 1:** Clinical characteristics of VaMCI patients and NC groups.

	**NC group (*n* = 32)**	**VaMCI group (*n* = 31)**	**χ^2^/*t***	***P*-value**
Sex (male/female)	18/14	18/13	0.21	1.000
Age (years)	62.72 ± 8.22	63.84 ± 14.1	−0.39	0.701
Education (years)	10.25 ± 2.33	9.32 ± 2.12	1.65	0.104
MoCA score	27.75 ± 1.72	23.32 ± 1.33	11.40	< 10^−4^
MMSE score	28.75 ± 1.39	26.32 ± 2.06	5.47	< 10^−4^
ADL score	20.00	20.87 ± 1.43	−3.39	0.001
HAMA score	1.69 ± 3.08	3.16 ± 3.66	−1.73	0.089
HAMD score	1.37 ± 3.18	2.61 ± 3.59	−1.45	0.152

*MoCA, Montreal Cognitive Assessment; MMSE, Mini-Mental State Examination; ADL, Activity of Daily Living scale; HAMA, Hamilton Anxiety Rating scale; HAMD, Hamilton Depression scale; values are means ± SD*.

### ReHo Values Differ Between VaMCI Patients and NCs

**Figure 1:**
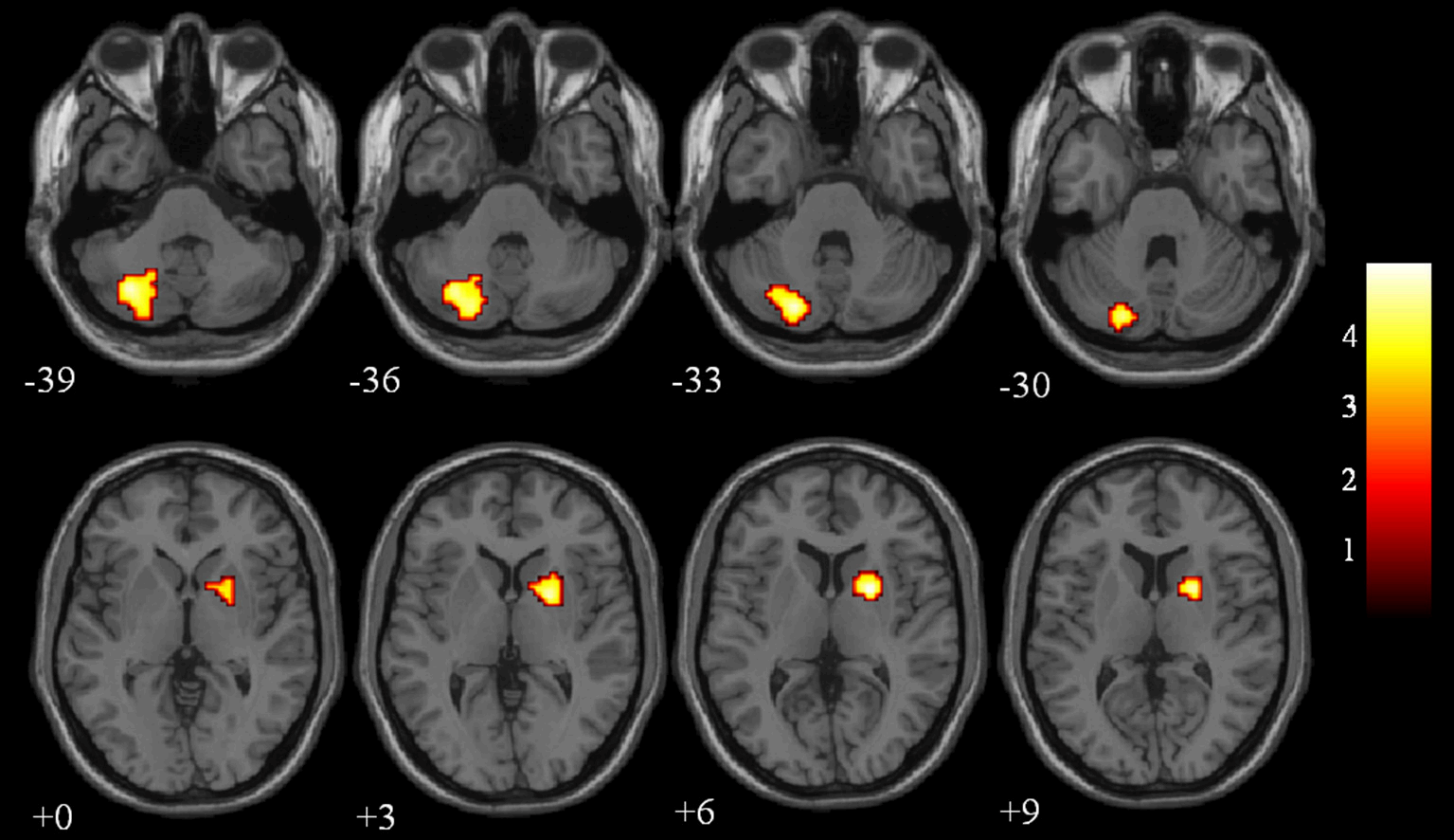
Between-group ReHo results were thresholded at a voxel-wise *P* < 0.05 (FWE corrected) and cluster size >10 voxels; color bar indicates t-score.

The differences in ReHo values between the NC and VaMCI group are shown in Figure 1 and Table 2. Compared to the NCs, the VaMCI patients showed a significant ReHo decrease in the right lentiform nucleus, left cerebelum_crus2 (*P* < 0.05 TFCE FWE corrected).

**Table 2:** ReHo results for the NC and VaMCI groups.

**Region**	**Cluster size**	MNI^*^			***T*-score**
	**(voxels)**	**x**	**y**	**z**	
**0.01–0.08**
Cerebelum_Crus2_L	201	−21	−78	−33	4.84
Lentiform_R	73	21	3	6	4.99

* *Numbers indicate the Z coordinate according to the Montreal Neurological Institute (MNI); between-group results were thresholded at a voxel-wise P < 0.05 (FWE corrected) and cluster size >10 voxels; L, Left; R, Right*.

### EC-FC Changes Between VaMCI Patients and NCs

**Figure 2:**
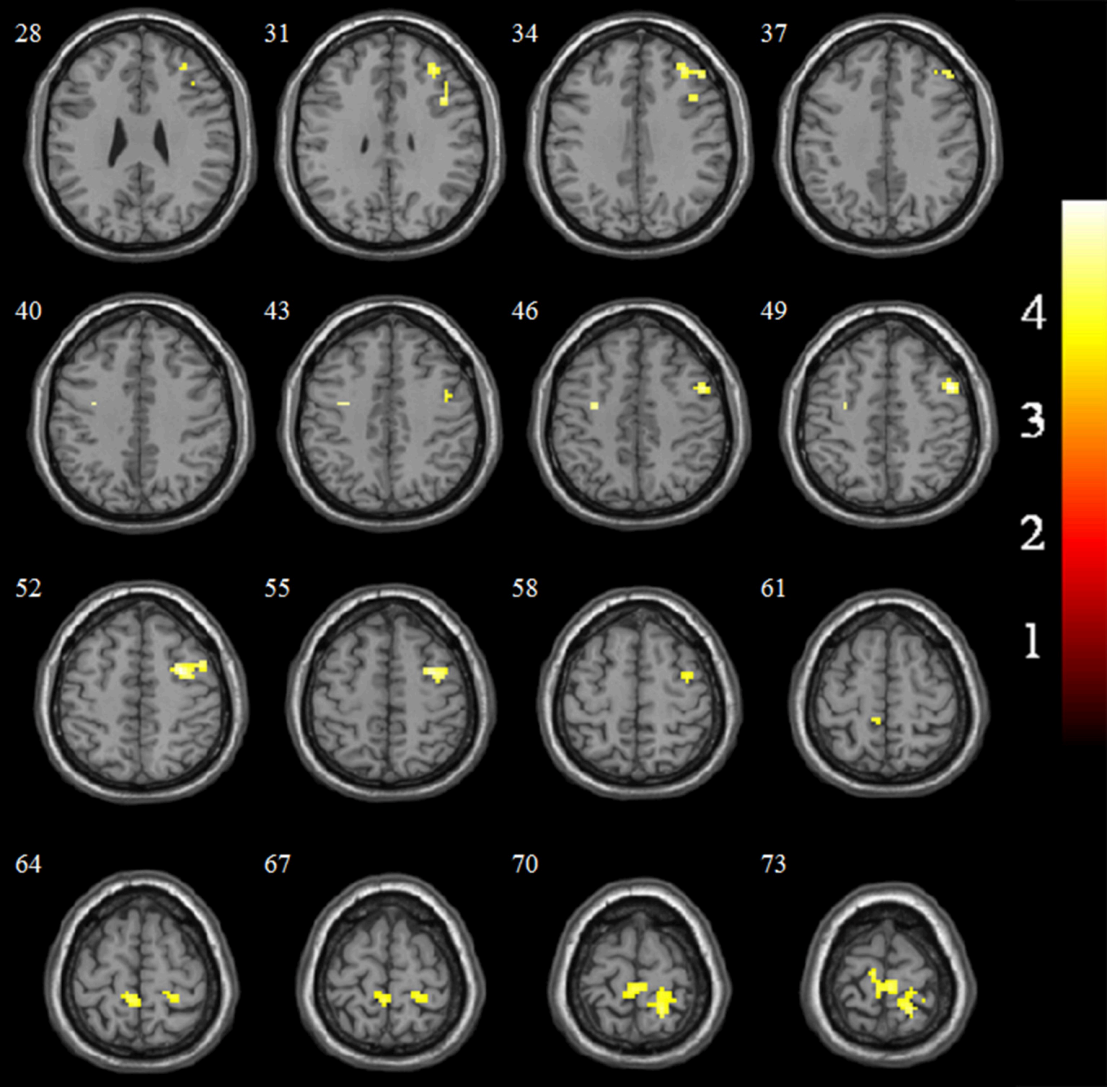
Significant EC-FC for the NC and VaMCI groups. *P* < 0.05 (FWE corrected) and cluster size >10 voxels; color bar indicates *t*-score.

The EC-FC changes between the NCs and VaMCI groups are shown in Figure 2 and Table 3. The VaMCI patients showed a significantly lower FC relative to the right EC and the right inferior/middle frontal gyrus, bitemporal pre-central gyrus, and right post-central/superior parietal lobule than the NC group (*P* < 0.05, TFCE FWE corrected).

**Table 3:** EC-FC changes for the NC and VaMCI groups.

**Region**	**Cluster size**	**MNI**			***T*-score**
	**(voxels)**	**x**	**y**	**z**	
**NC-VaMCI**
Frontal_Inf_R	13	42	15	30	4.01
Frontal_Mid_R	32	33	39	30	4.04
Precentral_L	10	−33	−6	42	4.08
Pre-central_R	74	48	6	48	4.63
Postcentral/Parietal_Sup_R	136	18	48	72	3.93

*Frontal_Inf_R, right inferior frontal gyrus; Frontal_Mid_R, right inferior/middle frontal gyrus; Pre-central_L, left pre-central gyrus; Pre-central_R, right pre-central gyrus; Postcentral/Parietal_Sup_R, right postcentral/superior parietal lobule*.

### Correlation Analysis

**Figure 3:**
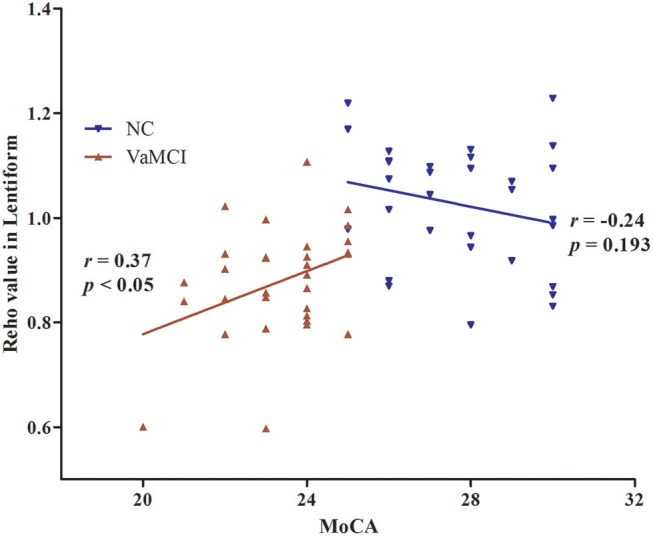
The linear correlation of ReHo values and MoCA scores in the right lentiform nucleus in VaMCI patients (*r* = 0.37, *P* < 0.05); red, VaMCI group; blue, NC group.

**Figure 4:**
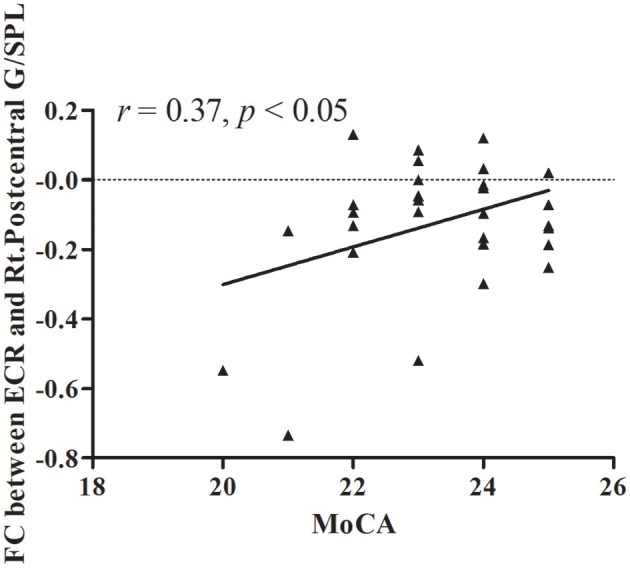
The linear correlation of FC between ECR and Rt.Postcentral G/SPL in VaMCI patients (*r* = 0.37, *P* < 0.05); ECR, right EC; Rt.Postcentral G/SPL, right postcentral/superior parietal lobule.

The results of partial correlation analyses indicated that the ReHo of the right lentiform nucleus positively correlated with MoCA scores in VaMCI patients (*r* = 0.37, *P* < 0.05) (Figure 3). There was no significant correlation between ReHo values and MoCA scores in the right lentiform nucleus in NCs (*r* = −0.24, *P* = 0.193) (Figure 3). In addition, the FC of the right post-central/superior parietal lobules showed a significant positive correlation between right EC-FC and MoCA scores (*r* = 0.37, *P* < 0.05) (Figure 4).

## Discussion

Previous studies have shown that normal brain development experiences local to holistic tissue patterns, which are important for revealing the neural mechanisms of cognition (24). Moreover, certain diseases, such as schizophrenia, relapsing-remitting multiple sclerosis, and type 2 diabetes mellitus, exhibit synchronicity changes in local functions that can also spread to distant brain regions (25–27). Our study shows that compared to NCs, patients with VaMCI had significantly lower ReHo in the left cerebellum and right lentiform nucleus and significant decreases in the FC of the right EC in the right inferior frontal gyrus, right middle frontal gyrus, bilateral pre-central gyrus, and right post-central/superior parietal lobule. The right lentiform nucleus showed a significant positive correlation between ReHo and MoCA scores and a significant positive correlation between the FC of the right EC and MoCA scores.

The cerebellum plays an important role in maintaining the balance of the body and coordinating its movements. An increasing number of studies have shown that the cerebellum also functions in cognitive regulation (28, 29). In their SPECT studies, Baillieux et al. found that the main cognitive function of the left cerebellum is attention and the function of visual space, confirming the cross-connection pattern of a loop passing from the cerebellum to the brain to the cerebellum again (30). Zheng et al. using BOLD-fMRI, found that both the cerebellum and cerebral cortex participated in the cognitive process of spatial memory (31). Similar to that study, we found that patients with VaMCI had significantly lower ReHo in the left cerebellum. Moreover, we also found that patients with VaMCI had significantly lower ReHo in the right lentiform nucleus. The lentiform nucleus which received the cholinergic projection of the Meynert basal forebrain and had extensive fibrous connections with the rest of the brain, playing a variety of functions such as motor coordination and cognitive activity. A wide range of diseases are related to the injury of the lenticular nucleus (bipolar disorder, schizophrenia, and so forth) (32, 33). As part of the basal ganglia, the lentiform nucleus is not only the core structure of the related motor system but also plays an important role in cognitive activities, such as working memory, executive function, learning, emotive behaviors, and reward. Cao et al. found a significant reduction in rCBF in the lentiform nucleus (34). In our study, the changes we observed in the ReHo of VaMCI patients imply that the left cerebellum and right lentiform nucleus were impaired; this is a pathophysiological foundation for the cognitive impairment evident in early stages of the disease.

Our study adds new evidence to the disconnection hypothesis because we investigated EC connectivity with all other brain regions. We found that several regions such as the right post-central/superior parietal lobule have disrupted connectivity to the right EC. In addition, Colby et al. using EEG, proved that the parietal lobe plays an important role in spatial memory (35). We found that VaMCI patients had significant decreases in the FC of the right EC in the right post-central/superior parietal lobule. This is related to the impairment of multiple cognitive domains such as orientation. In the frontal lobe region, we found that the lateral frontal regions (right inferior/middle frontal gyrus) showed disrupted connectivity to the right EC, which is related to attention processing (14). In addition, we also noticed that the bilateral pre-central gyrus showed decreased connectivity to the right EC. This may imply that the change in connectivity extended into the primary motor cortex in VaMCI patients. The relationship between EC and the above regions in VaMCI patients must be further investigated.

Based on correlation analyses, the decreased right lentiform nucleus showed a significant positive correlation between the ReHo and MoCA scores; there was a significant positive correlation between the decreased FC of the right EC connectivity with the right post-central/superior parietal lobule and MoCA scores. That is, the lower the FC, the lower the MoCA score. As noted above, the lenticular nucleus and the right post-central/superior parietal lobule play an important role in a variety of cognitive functions; these brain areas are also known to be the site of the pathophysiological foundations of cognitive impairment caused by the early stages of the disease.

As far as we know, our study is the first in which both ReHo and EC-FC analysis have been applied to rs-fMRI in VaMCI. We hypothesize that the ReHo and EC-FC would be disrupted in VaMCI patients. In theory, increased functional activity could provide a compensatory mechanism, through plasticity, helps limit the consequences of cognitive impairment. However, this putative compensatory function is at odds with what we have found in this study. These include the fact that no areas showed increased ReHo and EC-FC were detected in VaMCI patients than in the NCs. Hence, we consider that might be contribute to the light degree of cognitive impairment in the patients screened and the relatively small sample size, which requires more investigation (36).

There were some limitations to the current study. First, our sample size was small. Second, we could not exclude the interference of potential confounding factors such as artifacts of the respiratory and cardiac cycles, and so on. Third, we did not add the MCI case groups which the early stage of AD for further analysis. Fourth, we used neuropsychological measures that reflected overall cognitive function in correlation analyses; more detailed measures, particularly for specific cognitive domains, must be further investigated. The last, we were unable to observe dynamic changes in ReHo and EC-FC following the onset of VaMCI. A longitudinal study is needed.

## Conclusion

The patterns of changes in ReHo and EC-FC in VaMCI patients and the correlation with neuropsychological measures may be a pathophysiological foundation of cognitive impairment, which will aid the early diagnosis of VaMCI.

## Ethics Statement

The study was conducted under a research protocol approved by the Ethics Committee of the Second Affiliated Hospital of Shanxi Medical University. A written informed consent was obtained from all participants prior to the study.

## Author Contributions

MZ designed of the study and carried out data collection and wrote the manuscript. ML and DL screened the clinical data of the study. YX and JN completed the acquisition of functional imaging data. XZ sorted the data. XJ carried out data analysis. YH and YY contributed to conceptualization of the study and revision of the manuscript.

### Conflict of Interest Statement

The authors declare that the research was conducted in the absence of any commercial or financial relationships that could be construed as a potential conflict of interest.
